# Functional performance comparison between real and virtual tasks in older adults: A cross-sectional study

**DOI:** 10.1097/MD.0000000000009612

**Published:** 2018-01-26

**Authors:** Ítalla Maria Pinheiro Bezerra, Tânia Brusque Crocetta, Thais Massetti, Talita Dias da Silva, Regiani Guarnieri, Cassio de Miranda Meira, Claudia Arab, Luiz Carlos de Abreu, Luciano Vieira de Araujo, Carlos Bandeira de Mello Monteiro

**Affiliations:** a School of Arts, Sciences and Humanities of the University of São Paulo —EACH—USP, São Paulo, SP; b Postgraduate Program in Public Policies and Local Development, School of Sciences of Santa Casa de Misericordia de Vitoria, Vitoria, ES; c Laboratory of Design of Studies and Scientific Writing, ABC School of Medicine, Santo Andre, SP; d Department of Speech Therapy, Physical Therapy and Occupational Therapy, School of Medicine, University of São Paulo; e Paulista School of Medicine, Department of Cardiology, Federal University of Sao Paulo - UNIFESP, São Paulo, Brazil.

**Keywords:** computer tasks, older adults, timing coincident, virtual reality

## Abstract

**Introduction:**

Ageing is usually accompanied by deterioration of physical abilities, such as muscular strength, sensory sensitivity, and functional capacity, making chronic diseases, and the well-being of older adults new challenges to global public health.

**Objective:**

The purpose of this study was to evaluate whether a task practiced in a virtual environment could promote better performance and enable transfer to the same task in a real environment.

**Method:**

The study evaluated 65 older adults of both genders, aged 60 to 82 years (M = 69.6, SD = 6.3). A timing coincident task was applied to measure the perceptual-motor ability to perform a motor response. The participants were divided into 2 groups: started in a real interface and started in a virtual interface.

**Results:**

All subjects improved their performance during the practice, but improvement was not observed for the real interface, as the participants were near maximum performance from the beginning of the task. However, there was no transfer of performance from the virtual to real environment or vice versa.

**Conclusions:**

The virtual environment was shown to provide improvement of performance with a short-term motor learning protocol in a timing coincident task. This result suggests that the practice of tasks in a virtual environment seems to be a promising tool for the assessment and training of healthy older adults, even though there was no transfer of performance to a real environment.

**Trial registration:**

ISRCTN02960165. Registered 8 November 2016.

## 1 Introduction

According to the World Health Organization, the world's population of individuals over 60 years of age will increase from the current 841 million to 2 billion by 2050. Ageing is usually accompanied by deterioration of physical abilities, such as muscular strength, sensory sensitivity, and functional capacity, making chronic diseases, and the well-being of older adults new challenges to global public health.^[1]^ Decline in physical function, a common feature of older age, has important outcomes in terms of physical health related to quality of life, falls, health care use, admission to residential care, and mortality.^[1]^ In this regard, most older adults tend to have chronic illnesses, and a lower portion present several other conditions, with the understood probability of the onset of chronic and degenerative diseases, as well as neurological and musculoskeletal deficits.^[2]^

Despite functional loss, studies have shown that older adults have the ability to acquire new performance skills similarly to young adults.^[3,4]^ Thus, they can use this ability to minimize the conditions of age and improve new abilities. In this context, technology can be useful to promote better performance for this population. A new technology currently being used in research and practice application to help acquire new skills is virtual tasks, as they afford the possibility to stimulate the practice of motor and cognitive skills, leading to performance improvement and initiating older adults’ contact with modern technology.

Recently, intervention methods with virtual reality (VR) have been introduced, providing enjoyable tasks for older adults.^[1]^ VR and interactive video gaming have emerged as new approaches in the practice of skills over the last 10 years^[5]^ and allow various possibilities in different domains of performance improvement for daily tasks, since VR can simulate a real environment.^[6]^ According to Wang and Reid,^[7]^ using VR as a motor and cognitive tool can promote flexibility and control in task management, thereby increasing the likelihood of the transfer of skills acquired safely and efficiently. VR enables individuals to have virtual experiences that are similar to reality.^[8]^ Several tasks accomplish predetermined goals through technologically simulated scenes that the individuals must react to as if they were performing actions in reality.^[9]^

Although this technology has been used frequently with older adults, the evidence of VR effectiveness in this population is scarce in the literature.^[10,11]^ Few studies focus on older adults as the subject of evaluation,^[12,13]^ but the majority are associated with physical and cognitive changes with older adults used as a control group in studies conducted with individuals with neurological diseases, such as in post-stroke,^[14]^ Parkinson disease,^[15]^ and multiple sclerosis.^[16]^

Considering the scarcity of research and because of the potential of technological advances and the use of VR to improve performance, the present study aims to determine whether a virtual environment task allows the same performance when transferred to a similar task in a real environment for older adults. If virtual tasks can be learned and then transferred to a real environment, VR could likely be used as a tool for older adults who can learn new tasks with safety and fun provided by the virtual environment while enabling improvements in the transfer to real tasks.

It may seem simple to accomplish the task in a virtual environment and transfer to the real environment; however, there are caveats when generalising performance improvements in more natural environments (more real). In virtual environments, participants should pretend as if they are performing a specific task. Consequently, performance is often relatively abstract and directed to intangible objects. Therefore, it is likely that the virtual environment stimulates different spatiotemporal organization when compared with natural environments.^[16]^

To examine these issues, we used a VR computer programme (3D objects) that simulates the Bassin Anticipation Timer involving a coincident timing task widely used in different publications.^[16–18]^ The virtual task performed on the computer uses hand movement in front of the Kinect sensor (i.e., the participant performs a gesture) instead of pressing a button to complete the task as used in the real Bassin Anticipation Timer. The main concerns are whether the performance improves in a task performed in a virtual environment (more abstract, without physical contact) and if there is transfer to the task performed in a real environment (more concrete and with physical contact) and vice versa.^[16]^

The difference in interpretation of older adults when performing a virtual task without tactile feedback likely influences the performance in a coincident timing task. Tactile feedback may include the sensation of touch as well as the roughness, temperature, and surface friction characteristics associated with the touched object.^[19]^ The studies of Yano et al and Spence^[20,21]^ state that the use of tactile sensations enhances the sensitivity of other stimuli present in the same environmental interaction and provides an efficient communication channel. Other studies^[21–23]^ also point out that the presence of tactile monitoring directly influences spatial perception, decreasing the number of errors in movement as the users realise they are in a safe and controlled environment, since tactile feedback is present in daily life. The opposite supposedly happens when the task does not have tactile feedback (e.g., virtual task), since individuals present difficulties for experience in an unknown environment.^[16,17]^

Therefore, considering the use of VR tasks in older adults, 2 factors must be investigated: whether a task practiced in a virtual environment could promote better performance than the same task in a real environment, and if performing a task in a virtual environment could enable transfer to the same task in a real environment and vice versa. The answers could modify the learning tasks for older adults, and VR could be investigated as a practice of several tasks aimed at performance improvement and transfer to a real environment, providing new skills for older adults.

## 2 Methods

This study was approved by the Ethics Committee for Analysis of Research Projects of the FMABC (protocol no. 39396814.9.1001.0082), and all participants signed a written informed consent form. Sixty-five older adults of both sexes (12 males and 53 females) aged 60 to 82 years (definition used by the World Health Organization) with a mean age of 69.6 years (SD = 6.3) were included for participation in the study. Other inclusion criterira were healthy cognitive and motor conditions to understand and perform the required activity, which consisted of scoring more than 24 points on a mini-mental state examination administered before testing.

### 2.1 Material and apparatus

**Figure 1:**
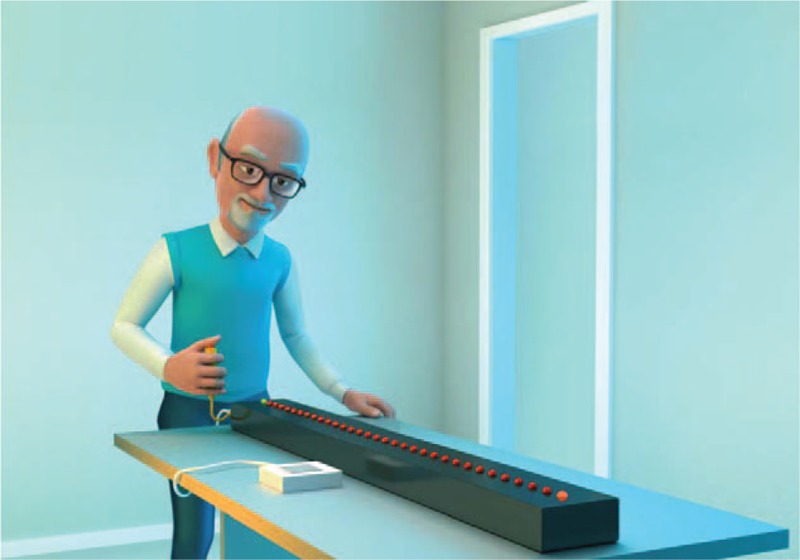
Coincidence timing task in the real environment, Bassin Anticipation Timer Model 35580.

We applied the timing coincident task to measure the perceptual-motor ability to perform a motor response in sync with the arrival of an external object at a certain point.^[24]^ This instrument has been widely investigated, especially in the motor learning area. In the study by Corrêa et al,^[25]^ different focuses were reviewed for timing coincident task use, including practice variability, stimulus speed, age, gender, level of complexity of the task, skill level, and results of knowledge. To evaluate the motor learning of the participants using a timing coincident task, 2 distinct interfaces were applied as follows:

Real environment interface (RI): The Bassin Anticipation Timer Model 35580 (Lafayette Instrument, Lafayette, Indiana) was used to represent the real environment, as used in several studies.^[25–31]^ This equipment was developed to test the area of visual acuity related to eye–hand coordination and anticipation. The participant was instructed to watch a light as it travels down the runway with 32 Light-Emitting Diode (LEDs) (2 runways with 16 red LEDs on each). A cue yellow LED was lit for 0.5 seconds after initiating a test and before the lights ran down the runway. The participant must anticipate the light reaching the target (last LED) and press a pushbutton to coincide with the arrival of the light at the target. The LCD readout displays the time difference between the response and the arrival of the light at the target and indicates if the response was early or late (Fig. 1).

**Figure 2:**
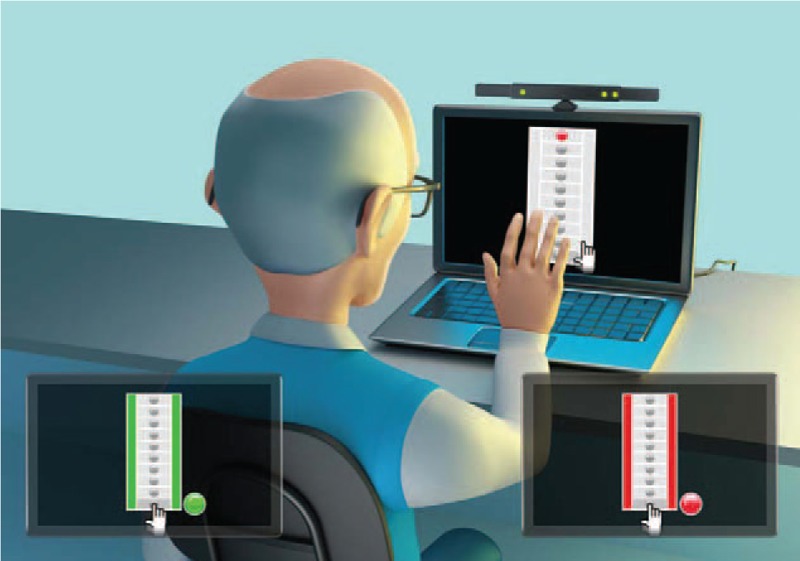
Coincidence timing task in the virtual environment. In the centre, the participant was waiting for the last bubble (target) to be lit. In the detail (left), the participant reached the target bubble. In the detail (right), the participant anticipated or delayed the target bubble.

Virtual environment interface (VI): We used a virtual coincidence timing task based on the Bassin Anticipation Timer developed by the Department of Electronic Engineering Polytechnic System,^[16,32]^ and updated by the Information Systems Laboratory of the University of São Paulo.^[17]^ In VI, 10 bubbles represented by 3D design on the computer are displayed simultaneously in a vertical column. The bubble lights change from grey to red sequentially (i.e., from the top to the bottom) until the target and last bubble (the 10th bubble).^[33]^ The task consists of a movement of the hand in a virtual environment produced by the Microsoft Kinect sensor at the moment the light reaches the target bubble, as proposed in RI task (Fig. 2).

### 2.2 Procedure and design

**Figure 3:**
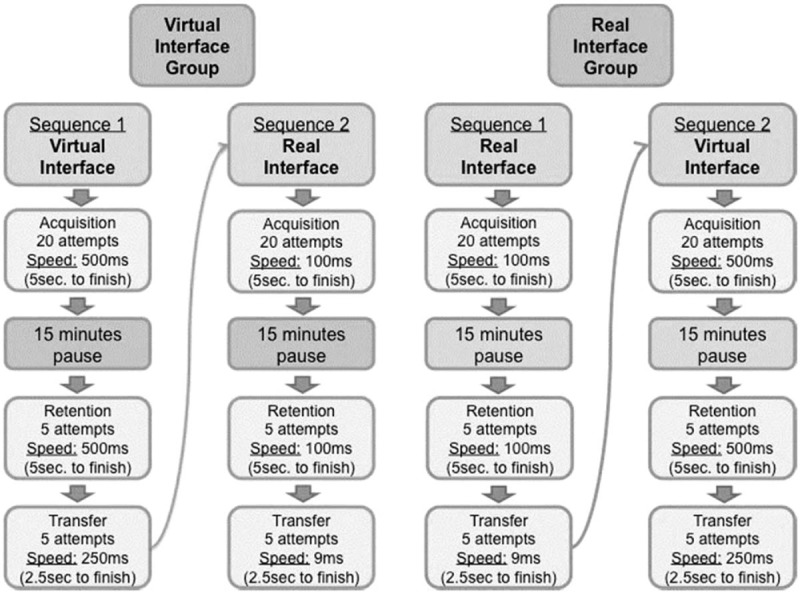
Study design.

The participants were divided into 2 groups: one group started in RI (RI group), and the other started in VI (VI group). Each participant used the dominant hand to perform the tasks (all right-handed). The task was performed in 20 trials for acquisition, 5 trials for retention, and 5 trials for transfer. Acquisition and retention were performed at the same velocity, and transfer with an increase of velocity. Both groups performed the tasks in the 2 interfaces, and prior to testing the order of the tasks was randomized and counterbalanced for each participant. The study design is presented in Figure 3.

The researchers provided a verbal explanation about the use of instruments before the participants started the tasks. The tasks according to the interfaces are described below.

#### 2.2.1 Virtual interface

Participants were seated on a chair in front of a computer (MS Windows 64 bits, Intel Core i7-4810MQ CPU @ 2.80 GHz) and Kinect sensor V1.0 (Fig. 2, centre) to facilitate and enable the task. The image of the participant was shown at the top right of the monitor, and his/her hand movement was shown on the main screen. The movement to reach the bubble was obtained by the Kinect sensor. The participant had to move his/her hand to pass over the target bubble. During acquisition and retention trials, the bubbles simulated a dropped light movement with the turning on and off of the lights at an interval of 500 ms (level 4) between position changes, while during transfer light the movement was increased 250 ms (level 5) between positions.

#### 2.2.2 Real interface

Participants stood in front of the Bassin Anticipation Timer, which was positioned vertically on a table (Fig. 1). The standing position was used in the real task to help the participant see the task with comfort. Participants were instructed that they should press the button when the target light was turned on (synchronously with the target light). The dropped light movement, with the turning on and off of the LEDs, occurred at an interval of 100 ms (l MPH) between position changes to acquisition and retention phases, and at an interval of 9 ms (11 MPH) to transfer.

Due to the difference in the number of LEDs (RI) and bubbles (VI) between the tasks, to equalise the protocol, the time between the start and end of the task was the same for all phases of the study (i.e., 5 seconds for acquisition and retention and 2.5 seconds for transfer).

### 2.3 Data analysis

Measuring the magnitude, bias, accuracy, consistency, and direction of response error is presumably the most common way of assessing performance and learning effects in behavioral research.^[34]^ Therefore, the dependent variables used in this study were the constant timing error (CE), absolute timing error (AE), and variable timing error (VE). Timing error is the time difference between the target LED and pressed button or target bubble and the registered hand movement.

CE, which is error resulting from a directional trend,^[35]^ is the temporal interval (in milliseconds) between the arrival of the visual stimulus and the end of the participant's motor response.^[36]^ The calculation was done by the simple arithmetic average of the error values, considering the algebraic sign (negative or positive) in a series of attempts (block of trials). It represents the direction of error if it is late or early.^[17]^ AE is the measure of performance accuracy, which is the estimator of the probability that an individual responds within a range around a target.^[35]^ It was calculated by taking the absolute value of each raw score, disregarding whether the response was early or late.^[36]^ VE is the error resulting from within-subject variability.^[35]^ It represents the consistency in a group of responses and is independent of the proximity of each trial to the designated target.^[34]^ The calculation was done by the square root of the sum of the square of difference between each score and the individual CE mean divided by the number of trials.^[17]^

The average was calculated for each of the 5 individual attempts, resulting in 4 acquisition blocks (A1, A2, A3, and A4), one retention block (R), and one transfer block (T). The dependent variables were submitted to 2 (sequence: RI first, VI first) by 2 (task: RI, VI) by 2 (block) Multivariate Analysis of Variance (MANOVA) with repeated measures on the last factor. For the factor block, separate comparisons were made for acquisition (first acquisition block A1 versus final acquisition block A4), retention (A4 versus retention block R), and transfer (R versus transfer block T). Post-hoc comparisons were carried out using the LSD (least significant difference) test (*P* < 0.05).

## 3 Results

### 3.1 Acquisition

The MANOVA revealed the following significant effects when comparing timing errors in the first acquisition block (A1) to errors in the final acquisition block (A4): main effects of task (Wilks λ = 0.183, *F*[3.59] = 87.5 *P* < .001, η^2^ = 0.82) and block (Wilks λ = 0.826, *F*[3.59] = 4.14, *P* = .010, η^2^ = 0.17). No interactions were found. The separate follow-up RM-ANOVAs for CE, AE, and VE are reported in the following sections.

### 3.2 Constant error

**Figure 4:**
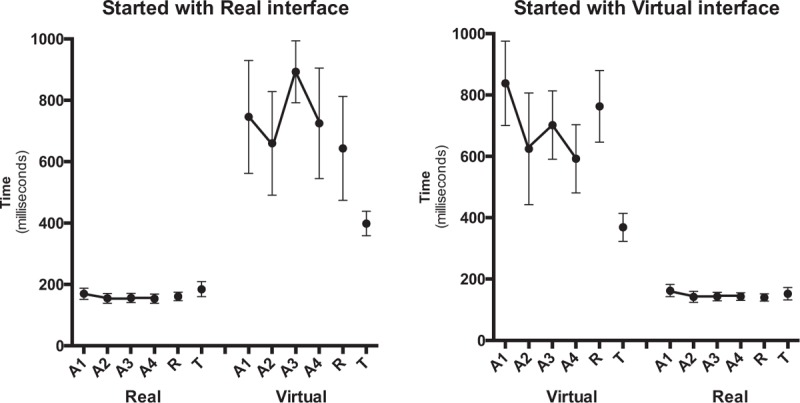
Constant error (ms) as a function of block and task for the group that started with a real task (left panel) and the group that started with a virtual task (right panel). A1–A4 = acquisition blocks, R = retention blocks, T = transfer blocks.

The repeated measures Analysis of Variance (ANOVA) for CE confirmed a significant effect for task, *F*(1.61) = 47.8, *P* < .001, η^2^ = 0.44. This result shows that all subjects responded significantly earlier (158 ms) in the real task compared to the virtual task (732 ms) (Fig. 4). All subjects presented a directional trend for delay.

### 3.3 Absolute error

**Figure 5:**
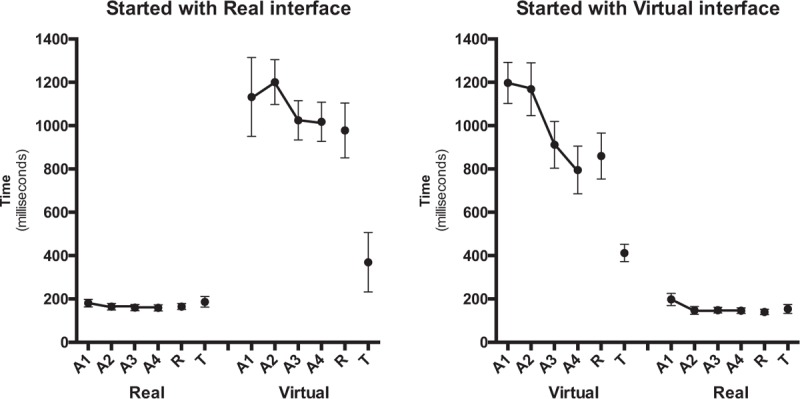
Absolute error (ms) as a function of block and task for the group that started with a real task (left panel) and the group that started with a virtual task (right panel). A1–A4 = acquisition blocks, R = retention blocks, T = transfer blocks.

The pattern of AEs is illustrated in Figure 5. The repeated measures ANOVA for AE showed a significant effect for task (*F*[1.61] = 128.5, *P* < .001, η^2^ = 0.68). This result indicated that the real task resulted in a significantly smaller AE (174 ms) than the virtual task (1025 ms). A main effect for block was found (*F*[1.61] = 6.39, *P* = .014, η^2^ = 0.10), indicating that the AE decreased from the first acquisition block (A1) (677 ms) to the final acquisition block (A4) (522 ms). However, the interaction was marginally significant for task by block (*F*[1.61] = 3.67, *P* = .060, η^2^ = 0.06), suggesting that this result can be due to the performance from A1 to A4 in the virtual task (1161–889 ms, respectively) and not in the real task (193–154 ms, respectively), according to the post-hoc test.

### 3.4 Variable error

**Figure 6:**
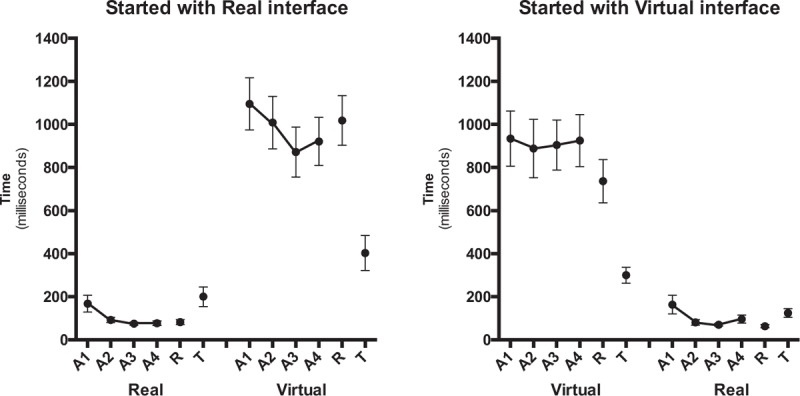
Variable error (ms) as a function of block and task for the group that started with a real task (left panel) and the group that started with a virtual task (right panel). A1–A4 = acquisition blocks, R = retention blocks, T = transfer blocks.

The pattern of VEs during acquisition is depicted in Figure 6. The repeated-measures ANOVA for VE confirmed significant main effects just for task (*F*[1.61] = 136.7, *P* < .001, η^2^ = 0.69). This result indicates that for a virtual task, the VE was much larger (940 ms) than for a real task (128 ms).

### 3.5 Retention

The MANOVA comparing the timing errors in the final acquisition and the retention blocks did not reveal any significant effect or interaction. There were no differences between the final acquisition block (A4) and the retention block for the constant, absolute, and variable errors (VEs; see Figs. 4–6). Since we are interested only in to what degrees practice effects were relatively permanent (i.e., differences between acquisition and retention), we do not report significant effects that do not involve the factor block (in fact, effects for task were similar to what is reported in acquisition).

### 3.6 Transfer

The MANOVA indicated differences in timing errors between the retention block and the transfer block (see Figs. 4–6). That is, a significant main effect for block was revealed (Wilks λ = 0.479, (*F*[3.60] = 21.7 *P* < .001, η^2^ = 0.52), as well as a significant block by task interaction (Wilks λ = 0.727, *F*[3.60] = 7.50, *P* < .001, η^2^ = 0.27). The separate follow-up RM-ANOVAs for CE, AE, and VE are reported in the following sections.

### 3.7 Constant error

The CEs during the retention block and the transfer block are shown in Figure 4. For the CE, there were significant effects of block (*F*[1.57] = 6.58, *P* = .013, η^2^ = 0.10) and block by task (*F*[1.57] = 10.2, *P* = .002, η^2^ = 0.15). Post-hoc comparisons indicated that the CE was smaller on the transfer block (285 ms) compared to the retention block (428 ms). This result, however, was due to the participants who performed the virtual task (i.e., 395 vs 698 ms) rather than the real task (175 vs 158 ms). All subjects presented a directional trend for delay.

### 3.8 Absolute error

Similarly to CE, there were significant effects of block (*F*[1.57] = 30.9, *P* < .001, η^2^ = 0.35) and significant interaction for block by task (*F*[1.57] = 29.2, *P* < .001, η^2^ = 0.34). Post-hoc comparisons indicated that the AE was smaller on the transfer block (270 ms) compared to the retention block (541 ms). This difference was a result of the performance of the participants who conducted the virtual task (T = 362 vs R = 922 ms), with no statistical difference for the participants who conducted the real task (T = 177 vs R = 159 ms). This means that all subjects had the capacity to transfer the task, with even better ability with higher speed for the virtual task (Fig. 5).

### 3.9 Variable error

The ANOVA with repeated measures for VE confirmed significant effects of block (*F*[1.57] = 34.9, *P* < .001, η^2^ = 0.38) and block by task (*F*[1.57] = 57.1, *P* < .001, η^2^ = 0.50). The VE was smaller in the transfer block (239 ms) than in the retention block (479 ms). Post-hoc further indicated that the decreased VE from the retention block to the transfer block occurred only for the virtual task (885–308 ms, respectively), while for the real task the VE increased significantly from the retention block (74 ms) to the transfer block (170 ms). For VE, a significant main effect was also found for sequence (*F*[1.57] = 4.16, *P* = .046, η^2^ = 0.68). The group that started the sequence in the virtual task had a smaller VE (306 ms) when compared to the group that started with the real task (412 ms).

## 4 Discussion

Considering the modernity and usefulness of virtual tasks to improve performance for older adults, one of the goals of the present study was to verify whether a task practiced using a VI could result in better performance than the same task in an RI. In this regard, there was improvement in the performance of the VI, but no change from the beginning to the end of the task in the RI. This result suggests that the participants were easily able to adapt themselves to the coincident timing task using the RI, and thus there was no improvement because they started reaching the maximum performance from the beginning. However, because the VI had higher difficulty, participants started with worse performance, which allowed improvement with practice (i.e., AE and VE). It is well known that improvement in performance is related to central changes, especially better forecasting and the anticipation of movement skills.^[37]^

In addition, regardless of the sequence of tasks (VI first or RI first), the results showed that the performance of both groups was better in the RI than in the VI. Older adults likely presented more difficulty during the virtual task because of the difficulties in dealing with technology without physical contact. Additionally, the performance in touching a real object (press the button) requires less difficulty in prediction of the object's movement, head control, and eye and arm movements. Moreover, in the virtual task using Kinect, the participants had to move their upper limbs, which required more strength, range of movement, and speed, which are commonly decreased in older adults, along with motor control and manual force.^[38]^

Difficulties faced by older people when using the VI are also present in daily life, as they have difficulties in performing some functional tasks that require more skills and adaptation, even to finish a fundamental activity in order to be independent.^[39]^ As proposed by Zimmerli et al,^[40]^ the main reason for reducing the speed of reaction and response in older adults appears to be a considerable decrease in the ability to process information, recognize, compare, and select according to an objective response.

The environment without physical contact, as provided by the Kinect system, probably created a complex situation for the participants, which hampered the flexibility of existing strategies with difficulties in performing the coincidence timing task. When a strategy does not work, due to a complex and challenging environment, people can choose to use an inefficient strategy,^[41]^ which could have caused difficulty for the virtual task. It is unclear how to predict the position of an object in a VI, and motor learning theories can help to provide clarification.

Motor learning depends on multiple processes, and each process is characterised by a motor learning rate that controls the memory and is updated strongly based on error and motion restraint.^[42]^ Besides improvement in the practice of the task in the acquisition phase, the retention and transfer tests were used to assess the motor learning process. The retention test was used to measure the capacity to maintain the same performance acquired with the practice after a period with no contact with the task, and it showed that in the short-term retention test for both tasks, all groups mantained the performance acquired in the acquisition phase. Thus, even considering the task in the VI, which seems to be more difficult, all participants improved their skill and retained their performance (infering motor learning).

When considering the transfer test, which assesses the ability to transfer the performance in a similar task or environment, evaluated by increasing the speed of the task, the results showed that in the virtual environment the performance of the older adults was better with the bubble dropping at a higher speed for both absolute and VEs. Different speeds were used as exercise progression to applied intervention with VR in older adults, and results showed increases in physical function, such as balance and gait velocity.^[43,44]^

Regarding the CE (i.e., evaluation of directional trend), the participants showed a tendency to delay the movement to achieve the task in both interfaces. Movement delay during a task in older adults is well estabilished and cited.^[45,46]^ According to Sterr and Dean,^[47]^ healthy ageing may affect the motor processes engaged in the anticipation and preparation of an expected response.

Our second objective was to verify whether the performance practiced in the VI enables transfer to the RI and vice versa. We analyzed the interference of the first sequence performed (i.e., virtual environment first or real environment first) in the second sequence. The results showed different task performances in both groups and no transfer between the environments. Although a coincident timing task was applied in both interfaces, pressing a button (RI) or making a movement in front of Kinect (VI) seems to be completely different for older adults.

In the learning process to perform a movement, the brain builds an association between self-generated motor commands and sensory feedback. An internal model is created and allows to predict the sensory consequences of self-generated motor commands.^[48]^ The characteristics of the practiced tasks in different environments as proposed in our study probably do not allow the creation of an effective internal model, and this internal model should enable to transfer the task.

Using advances in computing technology, VR task application seems to have potential to attend the daily challenges of older adults and can help assess and enhance cognitive and motor skills with various stimuli conditions not easily controllable or measurable in a real environment.^[49]^ However, there is a lack of data concerning the underlying neuronal mechanisms involved in motor learning and motor memory consolidation in older adults.^[50]^ Although several studies have suggested the potential of VR as a successful treatment and assessment tool in a wide variety of applications, VR tasks should be further investigated in motor and cognitive stimuli in older adults.^[37]^ Moreover, the VI and RI provide different information, and transfering performance between these environments should be considered cautiously.

## 5 Limitations

This study had some limitations. The first concern was the difference of stimuli in the real task (32 LEDs) and virtual task (10 bubbles). Although they are similar timing coincident tasks, it was not possible to create an identical virtual task with the same number of stimuli, which could have influenced the results. The second concern that could have influenced the results was the position of participants when performing the task: standing in front of the instrument in the RI and seated on a chair in front of a computer in the VI. Although it was possible to perform the task in the virtual environment while standing, we consider that the demand for execution of the movement would be much greater and could cause greater difficulty for the task. The third concern was the time processing of the game and relatively low sampling frequency (30 frames per second [fps] of the Kinect sensor), which could have influenced data accuracy. Although it depends on the application, some studies state that a rate of 24 fps is enough for images to come alive, thus avoiding perception of delays by the human visual system and, consequently, providing sensation of continuous movement.^[51]^ The fourth concern was the use of a short-term motor learning protocol. Future studies should assess the performance in both tasks in a long-term protocol, since tasks with no tactile feedback are uncommon in daily life and need more repetition to promote adaptation.

## 6 Conclusion

In summary, this study found that a virtual environment could be efficacious to provide short-term motor learning in a coincident timing task for older adults, as we found performance improvement in acquisition and retention as well as much higher performance in transfering to an increased speed. Regarding the 2 factors investigated in this study, the results showed that a task practiced in a virtual environment could not promote better performance than the same task in a real environment, and no transfer of performance was observed between real and non-immersive VR tasks in older adults, and transfering performance between these environments should be considered cautiously.
